# Supplementary material for: MetaRibo-Seq measures translation in microbiomes
Source: Nat Commun. 2020 Jun 29;11:3268. doi: 10.1038/s41467-020-17081-z (PMC7324362; doi:10.1038/s41467-020-17081-z)
Supplement: Supplementary file 10 — Supplementary Data 7 [file 41467_2020_17081_MOESM10_ESM.zip › File2/Confidence_VeryHigh_Taxonomy/178872_out.krona.html]

Javascript must be enabled to view this page.

members
magnitude
magnitudeUnassigned
count
unassigned
taxon
rank

178872\_out

26

26
2
superkingdom

phylum
976
26

class
26
200643

26
171549
order

26
171552
family

25
1283313
genus

species
25
671218

SRS012279\_contig\_number\_18861SRS015762\_contig\_number\_39331SRS018473\_contig\_number\_14798SRS019607\_contig\_number\_8959SRS024318\_contig\_number\_31581SRS024498\_contig\_number\_contig-100\_35450.35451SRS042643\_contig\_number\_contig-100\_147.141745.141745SRS047219\_contig\_number\_7959SRS053854\_contig\_number\_19800SRS054976\_contig\_number\_contig-100\_58.182007SRS076868\_contig\_number\_5933SRS076951\_contig\_number\_contig-100\_51.227346SRS077024\_contig\_number\_14049SRS077092\_contig\_number\_17470SRS078182\_contig\_number\_4766SRS097912\_contig\_number\_12719SRS098498\_contig\_number\_32340SRS103971\_contig\_number\_37351SRS143037\_contig\_number\_34698SRS143062\_contig\_number\_739SRS143354\_contig\_number\_12880SRS143450\_contig\_number\_15622SRS143529\_contig\_number\_contig-100\_22902.22902SRS147094\_contig\_number\_19881SRS147226\_contig\_number\_28955


SRS011115\_contig\_number\_42868
1
1852368
species
